# Meropenem and piperacillin/tazobactam prescribing in critically ill patients: does augmented renal clearance affect pharmacokinetic/pharmacodynamic target attainment when extended infusions are used?

**DOI:** 10.1186/cc12705

**Published:** 2013-05-03

**Authors:** Mieke Carlier, Sofie Carrette, Jason A Roberts, Veronique Stove, Alain Verstraete, Eric Hoste, Pieter Depuydt, Johan Decruyenaere, Jeffrey Lipman, Steven C Wallis, Jan J De Waele

**Affiliations:** 1 Department of Clinical Chemistry, Microbiology and Immunology & Department of Critical Care Medicine, Ghent University, De Pintelaan 185, Ghent, 9000, Belgium; 2 Department of Critical Care Medicine, Ghent University, De Pintelaan 185, Ghent, 9000, Belgium; 3 Burns Trauma and Critical Care Research Centre, The University of Queensland, Brisbane, Australia, Royal Brisbane and Women's Hospital, Herston, QLD 4029, Australia; 4 Department of Laboratory Medicine, Ghent University Hospital, De Pintelaan 185, Ghent, 9000, Belgium

**Keywords:** β-lactam antibiotics, hyperfiltration, kidney function, pharmacokinetics, pharmacodynamics, therapeutic drug monitoring

## Abstract

**Background:**

Correct antibiotic dosing remains a challenge for the clinician. The aim of this study was to assess the influence of augmented renal clearance on pharmacokinetic/pharmacodynamic target attainment in critically ill patients receiving meropenem or piperacillin/tazobactam, administered as an extended infusion.

**Methods:**

This was a prospective, observational, pharmacokinetic study executed at the medical and surgical intensive care unit at a large academic medical center. Elegible patients were adult patients without renal dysfunction receiving meropenem or piperacillin/tazobactam as an extended infusion. Serial blood samples were collected to describe the antibiotic pharmacokinetics. Urine samples were taken from a 24-hour collection to measure creatinine clearance. Relevant data were drawn from the electronic patient file and the intensive care information system.

**Results:**

We obtained data from 61 patients and observed extensive pharmacokinetic variability. Forty-eight percent of the patients did not achieve the desired pharmacokinetic/pharmacodynamic target (100% *f*T_>MIC_), of which almost 80% had a measured creatinine clearance >130 mL/min. Multivariate logistic regression demonstrated that high creatinine clearance was an independent predictor of not achieving the pharmacokinetic/pharmacodynamic target. Seven out of nineteen patients (37%) displaying a creatinine clearance >130 mL/min did not achieve the minimum pharmacokinetic/pharmacodynamic target of 50% *f*T_>MIC_.

**Conclusions:**

In this large patient cohort, we observed significant variability in pharmacokinetic/pharmacodynamic target attainment in critically ill patients. A large proportion of the patients without renal dysfunction, most of whom displayed a creatinine clearance >130 mL/min, did not achieve the desired pharmacokinetic/pharmacodynamic target, even with the use of alternative administration methods. Consequently, these patients may be at risk for treatment failure without dose up-titration.

## Introduction

Infection is a well-recognized but persisting problem in critical care medicine. Sepsis alone is the leading cause of mortality in non-cardiac intensive care units (ICUs), with up to 30% of patients dying within 1 month of diagnosis [1,2]. Adequate antibiotic therapy is one of the mainstays in treatment, with the emphasis on timely administration and appropriateness of the spectrum [3]. Optimizing antibiotic exposure is highly important as well, however, this is proving to be a greater challenge with recent data showing that antibiotic concentrations in critically ill patients are highly variable, unpredictable and commonly sub-optimal [4-7].

Antibiotic dosing regimens are usually determined in healthy adults with normal physiology or non-critically ill hospitalized patients. Both the volume of distribution and clearance are the key determinants of the pharmacokinetics of a drug. Unfortunately, pathophysiological changes in critically ill patients have a profound effect on both [8].

One of these pathophysiological changes is the development of augmented renal clearance (ARC). This is a phenomenon in which renal elimination of circulating molecules - including antibiotics - is enhanced. This, in turn, may lead to sub-therapeutic concentrations of time-dependent antibiotics such as β-lactam antibiotics, potentially causing therapeutic failure and selection of antibiotic-resistant pathogens. Critically ill patients are at risk for ARC, because of their pathophysiological disturbances, as well as the clinical interventions administered [9,10]. The incidence of ARC in critically ill patients is high and varies between 30% and 85% depending on the studied population and the definition of ARC [11-13].

One study has demonstrated the relationship between renal clearance and low antibiotic concentrations [14], but the relationship between renal clearance and β-lactam pharmacokinetic/pharmacodynamic characteristics has not been evaluated in a large cohort of patients. However, various pharmacokinetic modeling and simulation studies have suggested that using extended infusions will prevent low antibiotic exposure. However, this has never been tested in a large cohort of relevant patients with ARC. Therefore, the aim of this study was to assess the influence of renal clearance on pharmacokinetic/pharmacodynamic (PK/PD) target attainment when the antibiotic was administered as an extended infusion. Both the minimum target (50% *f*T_>MIC_), as well as the target of 100% *f*T_>MIC _which is considered to have higher bactericidal activity [15] were calculated. Notably this study enrolled patients without renal dysfunction, defined as an estimated glomerular filtration rate (eGFR) assessed by the MDRD equation of <80 mL/min.

## Materials and Methods

### Inclusion and exclusion criteria

The data used for this analysis were collected in two separate studies performed in the medical and surgical ICU of Ghent University Hospital, a tertiary care hospital with a total of 50 adult ICU beds. Both studies were approved by the Ethics Committee of the Ghent University Hospital (study 1: registration number 2009/543, study 2: 2010/814). Written informed consent was obtained from the patient or his/her legal representative.

Adult patients receiving either meropenem (Meronem^®^, AstraZeneca) or piperacillin/tazobactam (Tazocin^®^, Pfizer) were included if they did not meet exclusion criteria which included renal dysfunction (defined as an estimated glomerular filtration rate (eGFR) assessed by the MDRD equation of <80 mL/min/1.73 m²), absence of an arterial catheter, or absence of informed consent.

### Antibiotic administration

Patients received a loading dose (1 g meropenem or 4.5 g piperacillin/tazobactam) administered over 30 min, followed immediately by the first extended infusion dose of either antibiotic (1 g meropenem or 4.5 g piperacillin/tazobactam) every 6 h for piperacillin/tazobactam and every 8 h for meropenem. Extended infusion doses were administered over 3 h using a syringe pump via a central venous catheter.

### Sampling and β-lactam assay

The sampling strategy and β-lactam assay used was different in the studies that contributed patients for this analysis. Twenty patients were included in the first study, and 41 in the second.

#### Study 1 (20 patients)

Eight serial plasma concentrations were obtained from each patient between 24 and 48 h after the initiation of therapy at baseline and after 1, 1.5, 3, 3.5, 4, 6, and 8 h for meropenem; at baseline and after 1, 1.5, 3, 3.5, 4, 5, and 6 h for piperacillin. For each sample, 5 mL of blood was collected in heparin anticoagulant tubes without separator gel, via the arterial catheter. Specimens were centrifuged at 3,000 rpm for 10 min within 30 min of sampling, and then frozen at -80°C. They were shipped to the Burns, Trauma & Critical Care Research Centre of the University of Queensland, Australia for analysis by a specialized carrier.

The samples were analyzed at the Burns Trauma and Critical Care Research Centre, University of Queensland. The plasma concentrations of meropenem and piperacillin were determined by validated high performance liquid chromatography (HPLC) methods based on a published procedure that has been optimized for each drug [16]. Sample preparation was by protein precipitation with acetonitrile and a wash step with dichloromethane. Separations were performed on a Waters X-bridge C18 column (2.1 × 30 mm, 2.5 μm) with an acetonitrile: phosphate buffer mobile phase (pH 2.5 for meropenem, pH 3 for piperacillin). Detection was by UV at 304 nm (meropenem) or 210 nm (piperacillin). The meropenem assay was linear from 0.2 to 100 mg/L with an imprecision and inaccuracy <7% at high, medium, and low concentrations. The piperacillin assay was linear from 0.5 to 500 mg/L with an imprecision and inaccuracy <10% at high, medium, and low concentrations. Observed concentrations were corrected for protein binding (piperacillin 30%; meropenem 2%).

#### Study 2 (41 patients)

Two plasma samples were obtained per patient (mid-dose and trough), after administration of at least three doses, to ensure steady-state. For each sample, 5 mL of blood was collected in heparin-anticoagulant tubes without separator gel, via the arterial catheter. The samples were then sent to the core laboratory of the Dept of Laboratory Medicine at the Ghent University Hospital, where they were centrifuged and frozen immediately upon arrival at -20°C and were analyzed on the same day.

These samples were analyzed at the toxicology laboratory of the Department of Laboratory Medicine at the Ghent University hospital. The plasma concentrations of meropenem and piperacillin were determined by validated ultra high performance liquid chromatography coupled to tandem mass spectrometry (UPLC-MS/MS). Samples were deproteinized using acetonitrile. After centrifugation, a portion of the supernatant was diluted and injected on a Waters BEH C18 column (1.7 μm, 100 × 2.1 mm) kept at 50°C and a gradient elution of water and acetonitrile, both containing 0.1% formic acid. Compounds were detected with a Waters Acquity TQD mass spectrometer operating in positive electrospray ionization using a compound specific MRM method. The assay was linear from 2 to 80 mg/L for meropenem, and from 4 to 250 mg/L for piperacillin with an imprecision and inaccuracy <15% at high, medium, and low concentrations. Observed concentrations were corrected for protein binding (piperacillin 30%; meropenem 2%).

It should be highlighted that the samples in Study 1 and Study 2 were analyzed using different assays in two different laboratories. Although a formal inter laboratory validation was not undertaken, both methods have been independently validated according to FDA guidelines. Furthermore, both laboratories monitor the quality of their analysis by using internal quality controls at three levels.

### Pharmacodynamic analysis

Depending on the study and number of samples available, different methods were used to calculate the *f*T_>MIC_. When enough samples were available, the *f*T_>MIC _was calculated by observing the time during the dosing interval that the log-linear least squares regression analysis intersected the target MICs for *Pseudomonas aeruginosa *(16 mg/L for piperacillin and 2 mg/L for meropenem based on EUCAST breakpoints [17].

In the case when only two concentrations were available per patient, another approach was used. One concentration (C_1_) was taken halfway through the dosing interval, the second sample was a trough concentration (C_2_). Using these two concentrations, it is possible to calculate the elimination constant (equation 1).

(1) $${\text{C}}_{2}={\text{C}}_{1}-{\text{e}}^{\text{k }.\text{ t}}$$

Assuming one compartmental first order kinetics, this is sufficient to calculate the time within the dosing interval where the concentration reaches or drops beneath a certain threshold. In order to investigate if these two approaches are comparable, the *f*T_>MIC _for the samples from the first study was calculated using the pharmacodynamic analysis used for the second study. This was performed for validation purpose only and was not used for the analyses.

### Measurement of creatinine clearance and calculation of estimates

To calculate a reliable creatinine clearance, urine samples were taken from a 24-h collection. Creatinine was measured in both serum/plasma and urine using the rate blanked, compensated and uncompensated Jaffe technique, respectively (Modular P and Cobas 6000, Roche Diagnostics GmbH, Mannheim, Germany). The creatinine clearance was calculated as follows:

24-h creatinine clearance = U_v _× U_cr_/(1,440 × S_cr_), where U_v _is the urinary volume (mL), U_cr _the urinary creatinine concentration (μmol/L), and S_cr _the serum creatinine concentration (μmol/L).For assessment of ARC a cutoff of creatinine clearance ≥130 mL/min was used [14].

### Statistical analysis

The statistical analysis was performed using the statistical software package IBM-SPSS statistics version 20.0 (IBM Corp., New York, NY, USA). Data are expressed as median values with interquartile ranges (IQR) for continuous variables, numbers, and percentages for categorical variables. In order to identify important covariates, multivariate logistic regression analyses (single step, forced entry) were conducted with target attainment 100% *f*T_>MIC _and target attainment 50% *f*T_>MIC _as dependent variable using the variables which gave a *P *value of <0.10 in the univariate analysis. In the case of covariates which were closely related (such as weight, height, and BMI), the one with the most significant *P *value was chosen. Goodness of fit was assessed by the Hosmer-Lemeshow statistic. A receiver operator characteristic (ROC) curve was constructed to examine the sensitivity and specificity.

All tests were two-tailed, and *P *<0.05 was considered statistically significant.

## Results

### Patients

Sixty-one patients were included in the analysis. Patient characteristics on the day of study, and the comparison between the patients who did and did not reach the PK/PD target of both 100% *f*T_>MIC _and 50%*f*T_>MIC _are shown in Table 1. The median (IQR) creatinine clearance from all patients included in the study was 125 (93-173) mL/min ranging from 55 to 310 mL/min.

**Table 1:** Patient characteristics and comparison between patients who did and did not achieve the PK/PD target of 100% *f*T_>MIC _and 50% *f*T_>MI__C_. Data are reported as median (interquartile range).

Variable	All patients (*n*= 60)	PK/PD target (100% *f*T_>MIC_) achieved (*n*= 33/60) (55%)	PK/PD target (100% *f*T_>MIC_) not achieved (*n*=27/60) (45%)	*P *value	PK/PD Target (50% *f*T_>MIC_) achieved (*n*= 43/52) (86%)	PK/PD target (50% *f*T_>MIC_) not achieved (*n*=7/52) (14%)	*P *value
Male gender (*n*, %)	51 (85%)	28 (84%)	23 (85%)	0.721	36 (84 %)	7 (100%)	0.330

Age (years)	56 (48-67)	61 (53-73)	51(30-60)	0.016	60 (52-72)	48 (25-67)	0.054

Weight (kg)	78 (69-90)	75 (65-81)	83 (75-90)	0.014	75 (66-85)	85 (75-90)	0.041

Height (m)	1.75 (1.70-1.80)	1.75 (1.67-1.79)	179 (1.72-1.80)	0.170	1.74 (1.68-1.80)	1.79 (1.75-1.80)	0.098

BMI	25 (22-28)	24 (22-27)	25 (24-29)	0.084	24 (22-27)	25 (25-28)	0.188

SOFA at the day of study	5 (3-7)	5 (2-8)	5 (3-6)	0.693	5 (3-8)	4 (2-6)	0.358

Serum creatinine concentration (μmol/L)	54 (43-75)	53 (44-79)	56 (41 - 64)	0.623	57 (44-76)	54 (38-59)	0.306

Creatinine clearance (mL/min)		104 (87-123)	165 (138-208)	<0.001	106 (91-143)	215 (190-246)	<0.001

Antibiotic used Meropenem (*n*, %) Piperacillin (*n*, %)	17 (30%) 43 (70%)	7/17 (41%) 25/43 (58%)	10/17 (59%) 18/43 (42%)	0.24	9/11 (82 %) 33/41 (80 %)	2/11 (18 %) 8/41 (20 %)	0.515

SOFA: Sequential Organ Failure Assessment.

### Validation of the pharmacodynamic analyses

It was found that the results for both methods used for determination of *f*T_>MIC _were comparable.

### Creatinine clearance and PK target attainment

Sixty-one patients were included in the study. One patient was excluded from the analyses since no urine was collected, as a result of which the creatinine clearance could not be calculated. Six patients treated with meropenem had a trough concentration which was lower than the lower limit of quantification (2 mg/L), which is also the breakpoint MIC of *Pseudomonas aeruginosa*. This implies that these patients did not reach the desired target of 100% *f*T_>MIC_, but the exact % *f*T_>MIC _could not be calculated, as this is not possible using only one sample. Two patients treated with piperacillin/tazobactam could also not be used for this analysis, because only the trough concentration was available, which is not enough to calculate the exact % *f*T_>MIC _. These eight patients were included in the analysis using the PK/PD target of 100% *f*T_>MIC_, but could not be entered in the analysis using the PK/PD target of 50% *f*T_>MIC_.

#### Target 100% fT_>MIC_

Only 33 out of 60 patients (55%), for whom both creatinine clearance and trough concentrations were available, reached the PK/PD target of 100% *f*T_>MIC_. Patients who did not attain the predefined PK target (100%*f*T_>MIC_) were younger, had a higher creatinine clearance and a higher weight (table 1). Twenty-nine patients (48%) had ARC, of which 22 (76%) did not reach the PK target of 100%*f*T_>MIC_.

**Figure 1:**
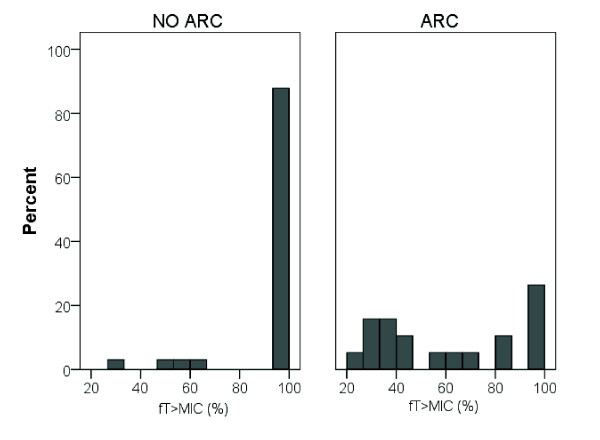
**Histogram *% f*T_>MIC _for patients with and without ARC**.

**Figure 2:**
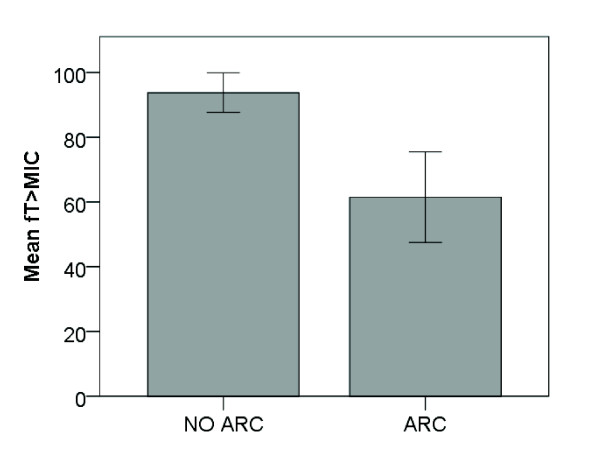
**Mean *% f*T_>MIC _for patients with and without ARC**.

Figure 1 illustrates the *f*T_>MIC _for the patients with and without ARC. The mean *f*T_>MIC _in patients with and without ARC is shown in Figure 2 and was 61% *vs*. 94% in patients with and without ARC, respectively (*P *<0.001).

**Figure 3:**
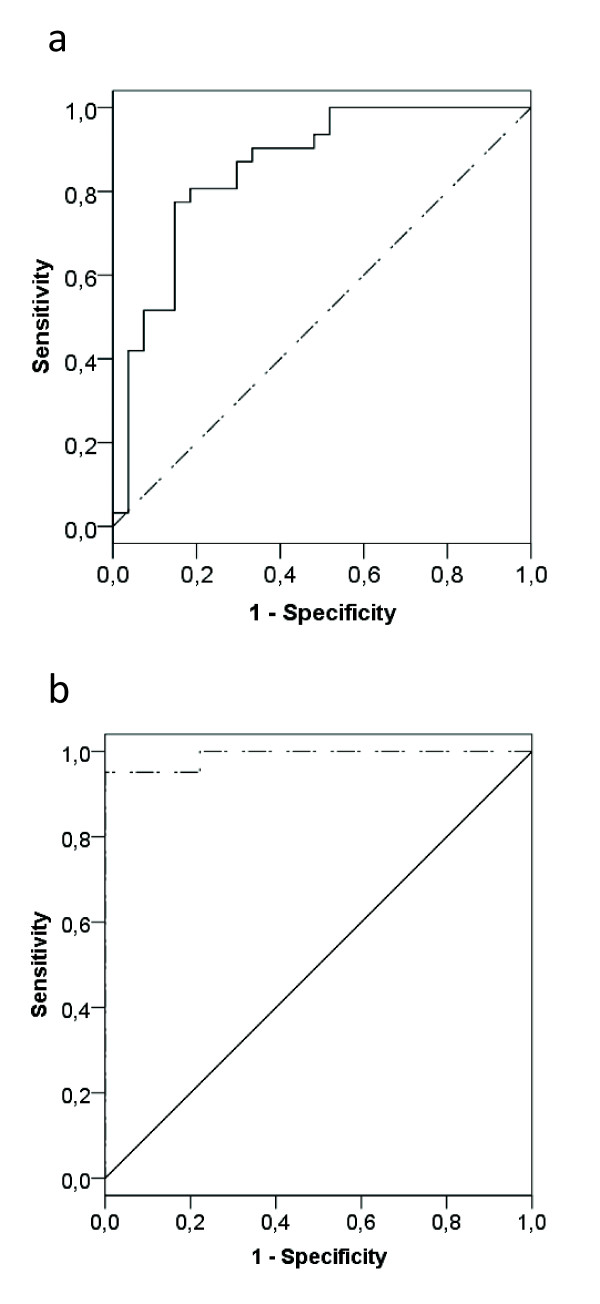
**ROC curves of the binary logistic model**.

The results of the multivariate logistic regression are shown in Table 2. As the antibiotic administered was not significantly different between the groups who did and did not achieve the PK/PD target, this was not included in the multivariate analysis (*P*=0.264). Contrary to creatinine clearance and weight, age was not found to be significant in the multivariate analysis. The area under the ROC curve was 0.86 (Figure 3a), with a sensitivity of 81% and a specificity of 81% for predicting target attainment at 50% probability.

**Table 2:** Multivariate regression model with attainment of 100% *f*T_>MIC _as dependent variable.

	Attainment of 100 % *f*T_>MIC _as dependent variable				
	
	B	*P *value	Exp(B)	95% CI for Exp(B)	
	
				Lower	Upper
Creatinine clearance (mL/min)	-0.028	0.002	0.972	0.955	0.990

Weight (kg)	-0.040	0.114	0.961	0.915	1.010

Age (years)	0.020	0.331	1.020	0.980	1.063

Constant	5.788	0.033	326.34		

**Figure 4:**
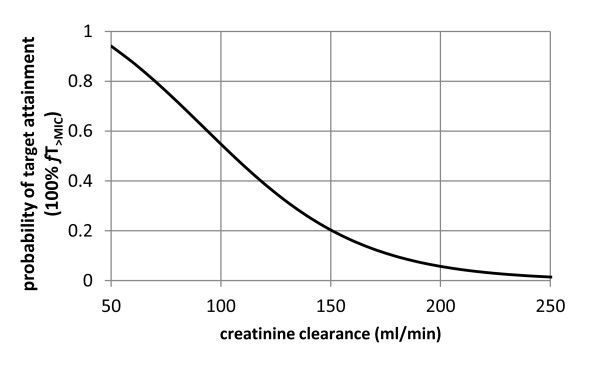
**Predicted probability of 100% *f*T_>MIC _target attainment**.

As an illustration of the impact of an increase in creatinine clearance, the probability of achieving the PK/PD target of 100% *f*T_>MIC _was plotted according to the creatinine clearance using the logistic model for a patient aged 55 years, weighing 75 kg (Figure 4).

#### Target 50% fT_>MIC_

Using the data from these 52 patients for whom both creatinine clearance and *f*T_>MIC _were available, we found that out of 19 patients displaying ARC, seven (37%) did not achieve the lower PK/PD target of 50% *f*T_>MIC _(*P *= 0.002) (Table 1).

The results of the multivariate logistic regression analysis are shown in Table 3. As the antibiotic administered was not significantly different between the groups who did and did not achieve the PK/PD target, this was not included in the multivariate analysis (*P*=0.515). The area under the ROC-curve was 0.99, with a sensitivity of 95% and a specificity of 100% for predicting target attainment at 50% probability (Figure 3b). Only creatinine clearance was found to be significant in the multivariate analysis.

**Table 3:** Multivariate regression model with attainment of 50% *f*T_>MIC _as dependent variable.

	Attainment of 50% *f*T_>MIC _as dependent variable				
	
	B	*P *value	Exp(B)	95% CI for Exp(B)	
	
				Lower	Upper
Creatinine clearance (mL/min)	-0.114	0.045	0.892	0.798	0.997

Weight (kg)	-0.035	0.616	0.965	0.841	1.108

Age (years)	0.005	0.906	1.005	0.926	1.096

Constant	24.07	0.07	2.8 × 10^10^		

## Discussion

In this large observational PK study, using clinical data from 61 critically ill patients with normal to increased renal function treated with meropenem or piperacillin/tazobactam, we found that ARC was associated with a higher risk of not achieving different PK/PD-targets in critically ill patients, even when administering these drugs by extended infusion. This calls into question the present approach to antibiotic dosing in these patients and supports use of more aggressive dosing strategies to minimize the likelihood of clinical failure.

In patients with apparent normal renal function, the relationship between creatinine clearance and low target attainment may not come as a surprise as previous studies have already demonstrated the correlation between creatinine clearance and clearance of β-lactam antibiotics [18-26]. However, to the best of our knowledge, this study is the first to report the association between creatinine clearance and the lack of attainment of different PK/PD targets including the lower target of 50% *f*T_>MIC _in patients with apparent normal renal function receiving antibiotic therapy administered as an extended infusion. Using trough antibiotic concentrations, Udy et al. have demonstrated the association between subtherapeutic β-lactam concentrations and creatinine clearance in select critically ill patients [14]. In the current study we could also investigate other targets as we were able to use data from the entire antibiotic infusion, including the lower PK-target of 50% *f*T_>MIC_. We found that, even when the dose was administered as an extended infusion, up to 37% of the patients with ARC did not achieve this minimum PK/PD target - and may thus be at risk for treatment failure.

Controversy exists in contemporary literature which PK target should be aimed for in critically ill patients, as it is not clear which PK/PD target is associated with highest probability of reaching clinical cure. Studies have shown that, depending on the antibiotic, 40% to 70% *f*T_>MIC _is necessary to treat infections [27]. However, recent research has shown that achieving higher targets may be associated with a higher probability of reaching clinical cure. In order to maximize the effect of β-lactam antibiotics, it may therefore be necessary to increase the *f*_T>MIC _to 100% or even maintaining the concentration four to five times the MIC for the entire dosage duration [28-30]. Nevertheless, irrespective of the PK/PD target considered relevant, increasing creatinine clearance is associated with lower target attainments.

Although ARC is a relatively new concept in intensive care medicine, its relevance should not be underestimated. The incidence in critically ill patients is high [11-13]. Implications for therapy with renally excreted drugs are considerable. Case reports have shown that some patients require up to 6, 8, or even 12 g meropenem per day to reach adequate serum concentrations [31,32]. The effects of renal clearance are important not only for β-lactam antibiotics, but have also already been described for other antibiotics, such as vancomycin [14,33].

This study has a number of limitations. First of all, this study did not look at clinical outcomes as the data were drawn from PK studies. Logically, clinical cure and mortality should be investigated in future validation studies of altered antibiotic dosing, although these studies should be even larger than the present study. Second, we have described renal function at inclusion using the MDRD which has been shown to underpredict glomerular filtration rate in some critically ill patients [34,35]. Moreover this study was only a snapshot, and might not be representative for the entire course of treatment as creatinine clearance varies in the course of the disease. Also, this study is a single-center study, which only included patients with apparent normal renal function, which limits extrapolation of these finding to all ICU patients. Finally, we have measured total drug concentrations with correction for protein binding based on literature. This is an oversimplification, but our data show that this approach is acceptable for these two antibiotics, although is not for more highly protein bound drugs.

The findings from this study suggest that an even more sophisticated method of optimization may be necessary in selected patients - patient-tailored antibiotic therapy - which is the adaptation of antibiotic therapy to the need of the individual patient in order to maximize efficacy and minimize toxicity through therapeutic drug monitoring and dose adaptation. Unfortunately, TDM of β-lactam antibiotics is currently challenging with long turnaround times, expensive equipment, logistical problems related to the instability of the antibiotics in the samples and the need for well-trained personnel. Efforts to overcome these limitations, and clinical studies to assess utility in the clinical setting are urgently needed [36].

## Conclusions

In conclusion, this study has demonstrated that in critical care patients receiving meropenem or piperacillin/tazobactam as an extended infusion, creatinine clearance is a key factor in the probability of PK/PD target attainment - irrespective if this is 50% or 100% *f*_T>MIC_. This study, which excluded patients with renal dysfunction, demonstrated that a specific subset of patients is at risk for PK/PD target non-attainment, more specifically those patients with increased creatinine clearances, even if the dose is administered as an extended infusion, which improves the *f*_T>MIC_. By means of multivariate logistic regression, it was found that a high creatinine clearance was an independent predictor of not achieving the PK/PD target, implying that without dose up-titration, these patients are at risk of treatment failure, even when extended infusions are used.

## Key messages

- Antibiotic concentrations vary greatly in intensive care patients with normal kidney function.

- The pharmacokinetic/pharmacodynamic target attainment is dependent on kidney function.

- Patients with augmented renal clearance have a high probability of target non-attainment, even with the use of an extended infusion strategy.

## Abbreviations

*% f*T_>MIC_: % time which the free fraction exceeds the minimal inhibitory concentration (MIC); ARC: augmented renal clearance; BMI: body mass index; BSA: body surface area; eGFR: estimated glomerular filtration rate; HPLC: high pressure liquid chromatography; k_e_: elimination constant; MDRD: modification of diet in renal disease; PK/PD: pharmacokinetic/pharmacodynamics; ROC: receiver operator characteristic; UPLC-MS/MS: Ultra high performance liquid chromatography coupled to tandem mass spectrometry

## Conflicts of interest

Dr Lipman is a consultant to AstraZeneca and Janssen-Cilag and has received honoraria from AstraZeneca, Janssens-Cilag, and Wyeth, Australia. Astra-Zeneca provides an annual donation to the Burns, Trauma and Critical Care Research Center. Dr Roberts has previously consulted for Astra-Zeneca, Janssen-Cilag, and Johnson and Johnson. The other authors have no conflicts of interest to declare.

## Authors' contributions

As principal investigator, MC had full access to all data in the study and takes responsibility for the integrity of the data and the accuracy of the data analysis. Study concept and design were performed by JDW. JDW, EH, PDP, and JDC carried out the coordination of the study. SW, VS, and AV were responsible for the laboratory analysis of the samples. Drafting of the manuscript was executed by MC and JDW. Critical revision of the manuscript for important intellectual content was done by JR and JL. All authors read and approved the final manuscript.
